# Autoimmune Atrophic Gastritis: The Role of miRNA in Relation to *Helicobacter Pylori* Infection

**DOI:** 10.3389/fimmu.2022.930989

**Published:** 2022-07-22

**Authors:** Fabiana Zingone, Valentina Pilotto, Romilda Cardin, Gemma Maddalo, Costanza Orlando, Matteo Fassan, Ilaria Marsilio, Eugenio Collesei, Filippo Pelizzaro, Fabio Farinati

**Affiliations:** ^1^ Department of Surgery, Oncology and Gastroenterology, University of Padua, Padua, Italy; ^2^ Gastroenterology Unit, Azienda Ospedale Università -Padova, Padua, Italy; ^3^ Department of Medicine, Surgical Pathology Unit, University of Padua, Padua, Italy; ^4^ Veneto Institute of Oncology, Istituto di Ricovero e Cura a Carattere Scientifico (IRCCS), Padua, Italy

**Keywords:** miRNA, autoimmune gastritis, gastric cancer, *Helicobacter pylori*, biomarkers

## Abstract

**Introduction:**

MicroRNAs (miRNAs) have been proposed as diagnostic markers, biomarkers of neoplastic progression, and possible therapeutic targets in several immune-mediated diseases. We aimed to analyze the expression profile of selected miRNAs (miR21, miR142, miR223, miR155) in patients with autoimmune atrophic gastritis (AAG), patients with non-autoimmune multifocal atrophic gastritis (MAG), and healthy control subjects (HC).

**Materials and methods:**

A total of 103 patients with AAG were consecutively recruited for this study among those attending our gastroenterology outpatient clinic. Participating patients were divided into two groups: primary, not Helicobacter pylori (HP)-associated related AAG (n=57, P-AAG) and HP-associated AAG (n=46, HP-AAG); this subgroup included HP-positive patients, patients with previously reported HP infection, and patients harboring antral atrophy, considered as a stigma of HP infection. We also included 20 sex-age-matched MAG patients and 10 HC. Upper endoscopy with gastric biopsies were performed on each AAG and MAG patient. Circulating levels of miR21-5p, miR142-3p, miR223-3p, and miR155-5p were measured by RT-PCR in all groups.

**Results:**

MiR-21 was over-expressed in P-AAG (p=0.02), HP-AAG (p = 0.04), and MAG (p=0.03) compared with HC. By contrast, miR-142 was more expressed in HC than in HP-AAG (p=0.04) and MAG (p=0.03). MiR-155 showed no significant differences among the four subgroups, while, unexpectedly, miR-223 was overexpressed in HC compared to P-AAG (p=0.01), HP-AAG (p=0.003), and MAG (p<0.001), and was higher in P-AAG than in MAG (p=0.05).

**Conclusions:**

MiR-21 was over-expressed in patients with gastric precancerous conditions irrespective of etiology, while in the same subgroups miR-142 and miR-223 were under-expressed compared to healthy controls. Controlling miRNAs up- or downregulation could lead to a breakthrough in treating chronic autoimmune diseases and potentially interfere with the progression to cancer.

## Introduction

Autoimmune atrophic gastritis (AAG) or chronic body-fundus atrophic gastritis, also known as type A gastritis, is a chronic, progressive inflammatory disease with immune-mediated, organ-specific pathogenesis (1). Its prevalence has been estimated to be ~0.5–4.5% globally (2). AAG prevails in females, with a 3: 1 ratio, and the risk increases in the presence of other autoimmune diseases (3–5). In a study analyzing 320 patients with AAG, an associated autoimmune disorder was present in 53.4%; the most common concurrent disease being autoimmune thyroiditis, found in 36% of patients (6).

While the exact pathogenesis of the disease has not been fully elucidated, in recent years, a relevant role for Helicobacter pylori (HP) infection has been demonstrated, this involving a molecular mimicry of some of the bacterial epitopes with the parietal cells proton pump and the production of cross-reactive antibodies with a proliferation of T cells showing a Th_1_ phenotype. To date, the hypothesis of a specific role of HP infection in AAG is considered insufficient “per se” to trigger the autoimmune disease. The involvement of a genetic predisposition (for instance, in HLA-related factors) and environmental factors are considered to be fundamental (7–9). On the other hand, HP is the main pathogenic factor for multifocal atrophic gastritis (MAG), which develops due to chronic gastric inflammatory damage elicited by the bacterium, starting from chronic non-atrophic gastritis (10). The Correa Cascade implies the linear progression of damage from chronic atrophic gastritis (CAG) with the development of a metaplastic intestinal epithelium (IM) to low-grade (LGD) and high-grade dysplasia (HGD) eventually to gastric adenocarcinoma (GC).

MicroRNAs (miRNAs) are a group of small single-stranded non-coding RNAs identified in numerous organisms. They originate from a stem-loop precursor, are transcribed by RNA polymerase II, and consist of 18-22 nucleotides. Their discovery is relatively recent, dating back to the early 90s, and their fundamental role is to regulate gene expression at the post-transcriptional level (11). They exert this role through recognition and pairing to specific sequences of mRNAs 3’untranslated Region (3’UTR), 5’ untranslated Region (5’UTR) Coding Sequence (CDS) with the final effect of messenger dysregulation, resulting in its down- and upregulation. However, pairing with the messenger is not perfect, which makes them able to regulate many genes (12). The participation of miRNAs in physiological and pathological processes has been clearly demonstrated: they play a role in cell proliferation, apoptosis, and differentiation and can therefore be involved in tumorigenesis, acting as tumor suppressors or oncogenes. Concerning the topic of this paper, recent literature has been published, in particular, underlining the role of miRNAs in the pathogenesis of gastric cancer (13–15). MiRNAs are also vital players in autoimmunity processes, regulating the immune response. Their dysregulation may, in turn, involve immune cells, including macrophages, monocytes, and dendritic cells. MiRNAs can also modulate adaptive immunity involving T and B cells through cytokine expression. Indeed, miRNAs have been thoroughly and exhaustively studied in many autoimmune diseases such as rheumatoid arthritis, multiple sclerosis, and diabetes mellitus (16).

MiRNAs have also been identified in extracellular fluids, mediate an autocrine and paracrine communicative function. Unlike cellular miRNAs, which are degraded in the extracellular environment within seconds, circulating miRNAs are extremely stable and survive long, even under unfavorable conditions (17).

With this in mind, we aimed to analyze the expression profile of some selected miRNAs (miR21, miR142, miR223, miR155) which are known to be involved in gastric cancer progression (15) in patients with AAG (either “primary” or HP-related), in patients with non-autoimmune MAG and healthy control subjects.

## Material and methods

### Study Population

AAG patients aged 18 to 65, followed up at Gastroenterology Unit of the Padua University Hospital, were consecutively enrolled in the study between January 2015 and April 2021. As comparison groups, we prospectively selected and included in the study sex and age-matched patients with MAG who had undergone upper gastrointestinal (GI) endoscopy in the last two years and sex and age-matched healthy controls (HC) from the hospital staff.

AAG and MAG patients were selected at the time of the first follow-up upper GI endoscopy performed during the study period. Subjects who took non-steroidal anti-inflammatory drugs (NSAIDs), or those who had malignancy, metabolic, or immunosuppressive disorders were excluded from the study. Each subject provided written informed consent to participate in the study, which was conducted according to the Declaration of Helsinki and was approved by the Ethics Committee of the Padova University Hospital (CESC approval 3312/AO/14 prot. 0034435).

The diagnosis of AAG, performed at least 6 months before the study enrollment, was based on the presence of anti-parietal cell antibodies, compatible histological findings and/or a compatible Gastropanel test (18) with higher than normal G17 or total Gastrin levels and lower than normal PGI and PGI/PGII values.

AAG patients were divided into two subgroups: “primary” AAG (P-AAG) and HP-related AAG (HP-AAG). The P-AAG group consisted of patients with an AAG diagnosis without any previous anamnestic or actual evidence of HP infection (at histology, anti-H.pylori antibodies, or the patient referred medical history) and no histological history evidence of antral atrophic changes, considered as a proof of previous HP infection. The HP-AAG subgroup was conversely composed of subjects with an AAG diagnosis based on the above criteria, together with previously verified evidence of HP positivity with subsequent eradication or histological evidence of antral atrophy, considered a stigma of the previous infection. Previous HP infection status was determined based on personal history, laboratory data (anti-HP antibodies), and histology (H&E, Alcian-PAS, and Giemsa for HP) (19).

The diagnosis of MAG, performed at least 6 months before the study, was confirmed by compatible histological findings (10), concurrent or previous reported H.pylori infection, absence of changes in the body, and fundic mucosa suggesting autoimmune gastritis and absence of anti-parietal cell antibodies.

### Endoscopic and Histological Evaluation

All patients (AAG and MAG) underwent an esophagogastroduodenoscopy (EGDS) with the standard updated Sydney System biopsy protocol (20) plus an extensive sampling of the gastric body/fundic area in patients with AAG and of the antral/incisura/lesser curvature in patients with MAG. The pathology samples, paraffin-embedded, were analyzed by an expert GI pathologist (MF) and scored based on the OLGA staging system (21, 22). For OLGA staging purposes, atrophy was assessed for loss of appropriate glands (i.e., intestinal metaplasia in antral and/or oxyntic biopsy samples); pseudo-pyloric metaplasia (PPM) in oxyntic biopsy samples. Glandular atrophy was scored as mild, moderate, and severe according to the recommendations of the OLGA staging tutorial (23). Enterochromaffin-like cell (ECL) hyperplasia was assessed according to internationally validated criteria, distinguishing linear vs. micro nodular, nodular, adenomatoid hyperplasia, and neuroendocrine tumor (NET). The extent of gastric inflammation (considered as lymphocytes/plasma cells infiltration) was expressed with a semiquantitative score from 0 to 3 (+, ++, +++).

### RNA Isolation and miRNAs Analysis

Immediately before the endoscopic procedure, 10 ml of peripheral blood were collected, and 5 mL were used for serum and plasma separation. Samples were preserved at -80°C until the assays of biochemical markers.

MiRNA analysis was performed in the plasma of all groups (AAG, MAG, and HC). Total RNA was extracted from 200 μl of plasma samples of the cases and controls using commercially available miRNeasy Serum/Plasma Advanced Kit (Qiagen - GmbH, Hilden, Germany), following manufacturer’s protocol, and finally dissolved in 20 ul of RNase-free water. Extraction efficiency was checked by adding synthetic oligonucleotides (UniSp2, UniSp4, UniSp5) at recommended concentrations.

According to the manufacturer’s instructions, reverse transcription for cDNA synthesis was performed using the miRCURY LNA RT kit (Qiagen).

The reverse transcription reaction system contained 4 μl of template RNA, 4 μl of miRCURY RT Reaction Buffer, 2 μl miRCURY RT Enzyme Mix, and 9 ul RNase-free water; samples were incubated for 60 min at 42°C, successively for 5 min at 95°C to inactivate the Reverse Transcriptase Enzyme and, finally, stored at -20°C.

Reverse transcription efficiency was checked by adding synthetic oligonucleotides (UniSp6). A miRCURY LNA miRNA PCR Assays and PCR Panels (Qiagen) was used for relative quantification of miR21-5p, miR142-3p, miR223-3p, and miR155-5p by qRT-PCR analysis. RT-PCR was performed on a PRISM 7900HT system (Applied Biosystems - Foster City, California, USA).

The reaction system contained 5 ul of SYBER Green Master Mix, 1 μl of PCR primer mix, 3 μl of cDNA template (60x diluted) and 1 μl of RNase-free water.

The cycling conditions for qRT-PCR were as follows: 95°C for 2 min, followed by 40 cycles of 95°C for 10 s and 56°C for 60 s and Melting curve analysis from 60 to 95°C.

The relative expression of each miRNA was calculated using the comparative cycle threshold (CT) method 2^-ΔΔCt^ (fold change) method, with miR93-5p, miR103a-3p, miR425-5p as internal controls for normalization. The expression levels of miRNAs were calibrated using the healthy volunteers as a reference control group.

### Data Analysis

Based on their distribution, continuous variables were expressed as means ± standard deviation (SD) or as median and 25°/75° percentiles, while categorical variables as absolute frequency and percentage. Possible differences between the two study groups were assessed with the independent-samples t-test or Mann-Whitney test for parametric and non-parametric variables, respectively. Stata 11 was used for statistical analysis. Statistical significance was set at p < 0.05 (2-tailed).

## Results

### Baseline Characteristics

A total of 103 patients with AAG, 20 MAG, and 10 HC were recruited during the study period. In the AAG group, the majority of patients were females (79.8%) with a mean age of 58.5 ± 12 years. The study was conducted on average 4.8 years (± 3.8 years) from the diagnosis of AAG. P-AAG subgroup included 57 patients, while the HP-AAG one included 46 patients. The two subgroups were similar in age and sex distribution (P-AAG 79% females, mean age 58 ± 11 and HP-AAG 74% females and 61 ± 13 years). A severe grade of inflammation in the gastric body mucosa was observed in six patients with HP-AAG and none with P-AAG (p=0.01). Finally, no differences were found in the presence and extent of intestinal metaplasia and ECL-Hyperplasia between the two subgroups. No differences in autoimmune comorbidities were found between P-AAG and HP-AAG, and overall the most frequent autoimmune condition was Hashimoto thyroiditis.

**Table 1** summarizes the AAG to MAG comparison. The AAG group, as expected, was associated with atrophy of the body, while in the MAG group, the atrophy of the antrum was obviously more common. However, when we considered the OLGA stage, we only found a statistically significant difference for stage IV that was only present in patients with MAG (p<0.001). Considering AAG group, an OLGA stage II was more frequently diagnosed in P-AAG (95%), while the OLGA stage III was detected only in HP-AAG (28%).

**Table 1:** Histological characteristics in AAG compared to MAG patients.

		P-AAG (n 57)	HP-AAG (n 46)	MAG (n 20)	*p-value*
Antral atrophy	0	57 (100%)	32 (70%)	0 (0%)	<0.001°°
	1	0 (0%)	14 (30.4%)	2 (10%)	
	2	0 (0%)	0 (0%)	16 (80%)	
	3	0 (0%)	0 (0%)	2 (10%)	
Body atrophy	0	0 (0%)	0 (0%)	9 (45%)	<0.001°°
	1	3 (5.3%)	6 (13%)	9 (45%)	
	2	11 (19.3%)	7 (15.2%)	0 (10%)	
	3	43 (75.4%)	33 (71.7%)	2 (10%)	
OLGA stage	I	3 (5%)	6 (13%)	2 (10%)	<0.001°°
	II	54 (95%)	27 (59%)	12 (60%)	
	III	0 (0%)	13 (28%)	4 (20%)	
	IV	0 (0%)	0 (0%)	2 (10%)	

°°Chi-square.

### miRNAs Analysis

**Figure 1:**
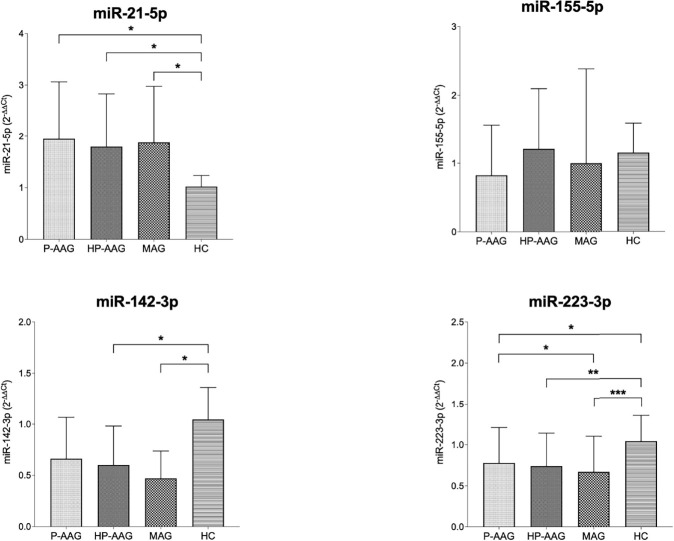
miRNA expression in our study population. *p < 0.05 and ≥0.01. **p < 0.01 and ≥0.001. ***p < 0.001.

As reported in **Table 2** and **Figure 1**, miRNAs levels were compared in patients with AAG, separately in those with P-AAG and HP-AAG, in patients with MAG, and in healthy controls (HC).

**Table 2:** miRNA expression in our study population.

	P-AAG(57/103)	HP-AAG(46/103)	MAG(n 20)	HC(n 10)	P-AAGvsHP-AAG	P-AAG vsMAG	HP-AAGvsMAG	P-AAG vsHC	HP-AAGvsHC	MAGvsHC
**miR 21-5p°**	1.95 ± 1.11	1.79 ± 1.03	1.87 ± 1.09	1.02 ± 0.21	0.47	0.80	0.77	**0.02**	**0.04**	**0.03**
**miR 155-5p^¥^**	0.82 (0.5–1.56)	1.21 (0.66-2.04)	1 (0.25-2.3)	1.15 (0.70-1.55)	0.10	0.62	0.60	0.52	0.62	0.95
**miR 142-3p°**	0.77 ± 0.43	0.74 ± 0.40	0.67 ± 0.43	1.04 ± 0.31	0.64	0.64	0.52	0.09	**0.04**	**0.03**
**miR 223-3p°**	0.66 ± 0.40	0.60 ± 0.37	0.47 ± 0.26	1.04 ± 0.32	0.43	**0.05**	0.17	**0.01**	**0.003**	**<0.001**

°mean ± SD (p=t-test), ^¥^median and 25°/75° percentiles (p=Mann-Whitney test). In bold, statistically significant results.

Overall, miR21 was significantly overexpressed in all pathological gastric conditions compared to HC. Conversely, miR142 was overexpressed in HC compared with HP-AAG (p=0.04), MAG (p=0.03), and P-AAG, even if, in this last case, a statistically significant difference was not reached (p=0.09). MiR155 levels showed no significant differences among the four groups. MiR223 was overexpressed in HC compared with P-AAG (p=0.01), with HP-AAG (p=0.003), and with MAG (p<0.001). Moreover, miR223 was more expressed in P-AAG than in MAG (p=0.05).

## Discussion

In the last decade, miRNAs expression has been correlated with gastric diseases. To better understand their expression and assess whether what was observed at tissue level was also confirmed in circulating blood, we compared the circulating levels in patients with P-AAG and HP-AAG and 20 patients with a histological diagnosis of MAG, and a group of 10 healthy subjects.

### miR21

MiR21-3p, known as making part of the group of onco-miRNA, is encoded by the miR21 gene on chromosome 17q23.2. It is overexpressed in numerous neoplasms, including breast and prostate cancer and tumors of the gastrointestinal tract, such as esophageal, gastric, and pancreatic cancer.

The data on its expression in precancerous gastric conditions, such as gastric atrophy, are more limited and concerns only its expression at the tissue level.

Link et al. (15) have documented a progressive increase in the expression of miR21 in biopsies obtained at the antral level in patients with non-atrophic gastritis, atrophic gastritis, and gastric cancer, thus showing a progressive increase in miR21 proceeding along the Correa cascade (24). Zhang et al. investigated the pathogenic role of miR21, demonstrating high levels of expression of miR21 in cancer and particularly increased levels of the miRNA in HP infection. In the gastric carcinogenic cascade sustained by HP, the increased expression of miR21 represents a cofactor linked to progression, possibly involved in modulating cell proliferation, inhibiting apoptosis, and in the stimulation of invasion and metastasis. Elevated tissue levels of miR21 also appear to be correlated with a poor prognosis and a risk of relapse (25).

In agreement with the quoted literature, we found that miR21 is more expressed in pathological precancerous conditions than in healthy controls.

In addition to the already known role of miR21 in cancer, the miRNA has been identified also as a regulator of various immune cells, playing a role in the loss of tolerance, antibody production, and autoimmune diseases development. MiR21 plays an essential role in T cell biology by intervening in T cell apoptosis and their activation and differentiation. Overall, no difference in miRNA21 circulating levels was detected between AAG and MAG, on the one hand, a P-AAG and HP-AAG on the other, thus indicating that 1. Any role miRNA may have in autoimmunity is less relevant with respect to its oncogene role and 2. that, at least considering our results from the “liquid biopsy” point of view, miR21 can be considered as a warning of the presence of a gastric precancerous condition, irrespective of its etiology.

### Mir142-3p

MiR142-3p exhibits higher expression levels in healthy controls than in the other groups of patients with atrophy, again with very little correlation with subtype and etiology. Few data on miR142-3p are currently available in the literature, but apparently, its role in carcinogenesis is of tumor suppressor, as demonstrated in lung cancer and colorectal cancer (26, 27). It shows the same properties in gastric cancer, and miR142-3p levels were found to be dramatically reduced in cancer compared to healthy tissue (28). It also modulates the response to treatment through an autophagic mechanism (29). The finding of reduced levels in patients with cancer seems to be confirmed in our study. The circulating levels of miR142-3p are reduced in patients with a precancerous condition such as gastric atrophy.

It is interesting to observe that, as observed for miR21, the reduction in the oncosuppressor miR142-3p is not significantly different between patients with P-AAG, HP-AAG, and MAG, suggesting that the etiology of atrophy does not influence the expression of miRNAs and how the atrophy, once established, leads to an imbalance of pro- and anti-oncogenetic factors, whatever its cause.

### miR155

Also, miR155 is involved in immunity and carcinogenesis, playing a dualistic role as an anti- or pro-inflammatory factor and as an oncogene or a tumor suppressor. This mechanism indicates, once again, how these biological pathways are not unique in the function of the miRNAs, thus underlying a complex regulatory network also influenced by the microenvironment (30). As already described, proceeding in the various stages of the Correa carcinogenic cascade, the levels of miR155 progressively increase until reaching the maximum expression in atrophy. MiR155 is also more expressed in the body than in the antrum without HP infection (31).

A role of miR155 in the development of T_regs_ has been demonstrated, although knockout studies reveal that it does not appear to be vital for a suppressive function (32). Mir155, however, is essential in the development of autoimmunity, acting as an accurate controller of the Th_17_/T_reg_ balance (33). Its overexpression preferentially induces a polarized response toward Th_1_ and Th_17_, and generally, the function of MiR155 is opposite to that of miR146a (34).

MiR155 plays a dualistic role: anti- or pro-inflammatory and oncogene or tumor suppressor. Its expression is influenced by several factors and the microenvironment, which could explain the lack of significant differences between the three groups we observed in our study. The highest values ​​of miR155-5p are expressed in patients with HP-AAG and MAG, with the difference not reaching statistical significance. The lack of significance of the data is probably linked to the fact that in our sample, no patient had an active HP infection but only a previous one.

### miR223

MiR223 is one of the most widely investigated miRNAs in gastric carcinogenesis. It is overexpressed in GC tissue (35) and in circulating body fluids, as confirmed by Fassan et al., which showed that miR223 was progressively higher at tissue levels, starting from atrophic gastritis to GC and also in the advanced (OLGA 3-4) vs. early (OLGA 1-2) atrophy stages. Also, plasma levels were significantly elevated in patients with GC compared with atrophic gastritis, but no differences were demonstrated between patients harboring gastric atrophy and patients with simple dyspepsia (36).

In our experience, miR223-3p had significantly higher circulating levels in HC than in the other subgroups and patients with P-AAG compared to patients with MAG. This behavior is not in agreement with the data reported in the studies published so far in the literature. It has been shown that miR223 increases at tissue level along the Correa cascade, with increased levels in gastric atrophy and cancer (31, 36).

Aalami et al. conducted a study on the serum levels of miR223, comparing 39 gastric cancer patients with as many healthy controls, finding an increase in the serum expression of miR223 in cancer patients (37).

In addition to its pro-oncogenetic role, miR223 also has an anti-inflammatory function, and like many other miRNAs, its function can vary depending on the microenvironment. This could be the first explanation for our discordant result. Finally, a systematic review published in 2015 highlighted how the circulating levels of miR223 can also be influenced by environmental factors such as smoking, pollution, and the use of some drugs such as anti-platelet and anti-inflammatory (38) that have not been investigated in our sample.

### Study Limitations

A limitation of our study is the small series of patients, especially for controls, in part justifiable with the main aim of the study finalized at evaluating the atrophic gastric pathway, a disease not particularly common, nor for HP-related atrophy, whose incidence is dropping, or for autoimmune atrophy, for which most patients go undiagnosed. Secondly, the lack of end-stage disease in cancer progression limits the final considerations since our data refer to precancerous changes. We cannot relate them to what happens in cancer but to previous results obtained by our group. Extrinsic limitations are represented by the lack of agreement on the normalization procedures for circulating miRNAs levels, their not clearly defined role, and the absence of previous data regarding AAG for miRNA detection. The latter is not an absolute limit since our study represents innovative research that opens to other studies on this topic.

## Conclusions

MiR21 was hyper-expressed in pathological gastric conditions, while miR-142 and miR223 were under-expressed compared to healthy controls. Only for miR223, which has a pro-oncogenetic role and an anti-inflammatory function, a difference between AAG and MAG is detectable. In contrast, no difference seems to be present between P-AAG and HP-AAG in any miRNA expression.

Many pathways are potentially targeted by miRNAs. Overall, in our experience the selected miRNAs may be useful as biomarkers of the presence of gastric precancerous conditions, as a liquid biopsy, but do not differentiate its etiology. The possibility of controlling their up- or downregulation could lead to a breakthrough in the treatment of chronic diseases, in this scenario atrophic gastritis, and potentially interfere with cancer development. Developments in this field could open new therapeutic perspectives in the management of atrophic gastritis: the regression of atrophy and/or intestinal metaplasia could prevent neoplastic progression toward gastric adenocarcinoma.

## Data Availability Statement

The raw data supporting the conclusions of this article will be made available by the authors, without undue reservation.

## Ethics Statement

The studies involving human participants were reviewed and approved by Comitato Etico per la Sperimentazione Clinica di Padova. The patients/participants provided their written informed consent to participate in this study.

## Author Contributions

FZ and VP performed the research; GM, IM, EC, CO and FP collected the data; FZ analyzed the data; FF designed the research study; FZ wrote the first draft of the paper; MF and RC contributed to the design of the study. All authors revised and approved the final version of the article.

## Funding

MF is supported by grants from the Italian Health Ministry/Veneto region research program NET-2016-02363853 and AIRC 5 per mille 2019 (ID. 22759 program). This research was in part supported by the Department of Surgery, Oncology and Gastroenterology, University of Padua with the project titled: “The role of immune-inflammatory mechanisms, miRNA and gastric microbiome in the development of autoimmune atrophy neoplastic complications” (BIRD Budget 2021).

## Conflict of Interest

FF received commercial research funding from Gilead and Bayer, MF received commercial research funding from Astellas Farmas and QED. FZ received commercial research funding from Takeda, Janssen, EG, Sofar, Norgine.

The remaining authors declare that the research was conducted in the absence of any commercial or financial relationships that could be construed as a potential conflict of interest.

## Publisher’s Note

All claims expressed in this article are solely those of the authors and do not necessarily represent those of their affiliated organizations, or those of the publisher, the editors and the reviewers. Any product that may be evaluated in this article, or claim that may be made by its manufacturer, is not guaranteed or endorsed by the publisher.
